# The effect of transpyloric enteral nutrition on inflammatory response and prognosis for patients with Corona Virus Disease-19 in intensive care unit: A STROBE compliant study

**DOI:** 10.1097/MD.0000000000031294

**Published:** 2022-11-04

**Authors:** Wei Zhu, Ping Chen, Ke Wang, Xiaolei Xing

**Affiliations:** a Intensive Care Unit, Tianyou Hospital Affiliated to Wuhan University of Science and Technology, Hubei Province, China; b Endocrine Department, Tianyou Hospital Affiliated to Wuhan University of Science and Technology, Hubei Province, China.

**Keywords:** COVID-19, inflammatory response, NLRP1, TEN

## Abstract

To investigate the effect of transpyloric enteral nutrition (TEN) on NLRP1, inflammatory response and prognosis for patients with Corona Virus Disease-19 (COVID-19) in intensive care unit (ICU). The present prospective observational study included 29 cases of COVID-19 patients in ICU who admitted to our hospital during February 2020 to March 2020. All the patients were divided into gastrogavage groups (n = 16) and TEN group (n = 13) according to route of enteral nutrition. Serum levels of C-reactive protein (CRP), interleukin-1 β (IL-1β), interleukin-6 (IL-6), tumor necrosis factor-α (TNF-α) and NLRP1 (NLR family pyrin domain containing 1) was detected by enzyme linked immunosorbent assay (ELISA). Serum levels of lymphocyte, albumin and hemoglobin was detected using an automatic biochemical analyzer. Patients’ demographic and clinical characteristics were collected and analyzed. Kaplan–Meier (K-M) curve was conducted for survival analysis and receiver operating characteristic curve was used for the analysis of diagnostic value of biomarkers. All the patients were followed-up for 3 months. This study found that the survival group had higher rate of TEN therapies than the deceased. COVID-19 patients in ICU on TEN had lower APACHE II scores, frequency of feeding suspension and mortality, however, with higher content of albumin was found at 5th day. The incidence of nutritional intolerance including abdominal distension and gastric retention in patients on TEN was notably lower than those on gastrogavage. The serum levels of NLRP1, CRP, IL-1β, IL-6 and TNF-α decreased in a time-dependent manner, but patients on TEN had lower levels of NLRP1, CRP and IL-1β than patients on gastrogavage. A positive correlation was found among NLRP1 and inflammatory factors, and COVID-19 patients with lower NLRP1 had longer survival time. Serum NLRP1 also exhibited diagnostic value for the death of COVID-19 patients. TEN decreased inflammatory response and improved the prognosis for COVID-19 patients in ICU.

## 1. Introduction

Coronavirus disease 2019 (COVID-19) is a newly emerged respiratory disease caused by severe acute respiratory syndrome coronavirus 2 (SARS-CoV-2), with a mortality of approximately 3.7%.^[1]^ Accumulating evidences show that around 15% COVID-19 patients would progress to severe pneumonia with a cytokine storm syndrome and approximately 5% will eventually develop into acute respiratory distress syndrome (ARDS), septic shock or multiple organ failure.^[2,3]^ Therefore, it’s important to attenuate inflammatory response and decrease the mortality of COVID-19 patients.

Currently, various inflammatory cytokines function as the predictors for the disease severity in COVID-19 patients. Biomarkers such as C-reactive protein (CRP),^[4]^ interleukin (IL)-8,^[5]^ interleukin-10 (IL-10)^[6]^ and tumor necrosis factor (TNF)-α^[7]^ are all considered as diagnostic biomarkers for COVID-19 patients in clinic. As reported, the levels of interleukin-1β (IL-1β), IL-6, IL-8 and sTNFR1 were upregulated in COVID-19 patients, and obvious elevations of IL-1β, IL-6 and sTNFR1 and decreased IL-10 were found in COVID-19 patients in intensive care unit (ICU) than patients with community-acquired pneumonia.^[8]^ Valle et al also found that serum levels of IL-6 and tumor necrosis factor-α (TNF-α) were independent and significant predictors of disease severity and death for COVID-19 patients.^[9]^ The activation and assembly of NLRP1 (NLR family pyrin domain containing 1) was closely associated with inflammatory response. NLRP1 knockdown blocked the activation of Casp1 and Casp6 in intraneuronal inflammation.^[10]^ The inhibition of NLRP1/ASC inflammasome attenuated inflammatory response in fructose-induced retinal ganglion cells, suggesting NLRP1 inflammasome might be a potential biomarker against diabetic retinopathy.^[11]^ However, up to now, limited researches focused on the role of NLRP1 and inflammatory cytokines in COVID-19 patients on transpyloric enteral nutrition (TEN).

Malnutrition is very common for critically ill patients and increases the risk of complications and mortality for them. Enteral nutrition is the most common model of feeding for in patients ICU because it can stimulate immune system and reduce side effects, including the incidence of bacterial translocation, sepsis and multisystem failure.^[12]^ However, patients in ICU often show poor tolerance for enteral nutrition by oral or gastric route, TEN becomes a useful alternative for critically ill patients at risk of pulmonary aspiration or those with poor toleration of oral or gastric feeding.^[13]^ An analysis of transpyloric feeding and gastric feeding also shows that TEN decreases the incidence of pneumonia and improves nutritional status in patients with severe traumatic brain injury.^[14]^ However, the role of TEN in COVID-19 patients in ICU was not clearly illustrated in previous studies.

In the present study, we aimed to investigate the effect of TEN on inflammatory response and prognosis of COVID-19 patients in ICU. This study might bring more clinical evidences for the use of TEN and the effect of NLRP1 on COVID-19 patients in ICU.

## 2. Methods

### 2.1. Patients

The present prospective observational study analyzed the data of 39 COVID-19 patients in ICU who were admitted to our hospital during February 2020 to March 2020. A total number of 29 patients were finally enrolled in this study. All the patients were diagnosed as COVID-19 according to real-time reverse-transcriptase-polymerase chain reaction (rRT-PCR) and CT examinations. The diagnosis of severe or critical patients was depending on New Coronavirus-Infected Pneumonia: Severe and Critical Diagnosis and Treatment Program (Second trial version) formulated by the National Health Commission of China. Inclusion criteria of ICU patients: respiratory failure occurred and mechanical ventilation required; shock occurred; and organ failure. The following patients were excluded: there are contraindications of enteral nutrition such as gastrointestinal bleeding; the duration of ICU treatment was less than 48 hours due to the endangered status or other reasons; the cause of death was not directly related to the COVID-19, and lost in the follow-up. The enrolled 29 patients showed indications for nutritional therapy with NRS2000 > 3. All the patients were divided into gastrogavage group (n = 16) and TEN group (n = 13) according to route of enteral nutrition. Written informed consent was obtained from all patients. The present study was approved by the Ethic Committee of Tianyou Hospital Affiliated to Wuhan University of Science and Technology. Declaration of Helsinki was obeyed in this study.

### 2.2. Enzyme linked immunosorbent assay (ELISA)

Peripheral venous blood samples were collected at admission, 3 days and 7 days. Serum levels of CRP, IL-1β, IL-6 and TNF-α was detected by ELISA using commercially kits: Human IL-1β ELISA Kit (ab46052), Human IL-6 ELISA Kit (ab229434), Human TNF-α ELISA Kit (ab229399) (all purchased from Abcam, Cambridge, MA, USA) and human CRP ELISA Kit (#MBS3800117, MyBioSource, Inc., San Diego, CA, USA).

### 2.3. Data collection

Demographic data including age, sex, route of enteral nutrition and frequency of feeding suspension due to complications. Serum levels of lymphocytes, albumin and hemoglobin at 3rd and 5th day after the admission were detected using an automatic biochemical analyzer. Acute Physiology and Chronic Health Evaluation II (APACHE II) score was used to determine the severity of the illness. All the patients were followed-up for 3 months from admission to the last follow-up or failure.

### 2.4. Statistical analysis

Chi square analysis was used for comparison of counting materials and rates. Continuous data were presented by mean ± SD. Comparisons for two groups were conducted using student-*t* test, and comparisons among three or more groups were performed by using one-way analysis of variance (ANOVA) followed by Tukey post hoc test. Kaplan–Meier (K‐M) curve was conducted for survival analysis with the log-rank test. Receiver operating characteristic curve (ROC) was used for the analysis of diagnostic value of biomarkers. A *P*-value less than .05 was considered statistically significant. All calculations were performed using SPSS 20.0.

## 3. Results

### 3.1. Basic characteristics of all patients

A total number of 29 COVID-19 patients in ICU were included in this study. As shown in Table 1, no significant difference was found in age, sex, underlying disease and the serum levels of lymphocyte, albumin and hemoglobin between deceased and survival group. However, the rate of TEN was much higher in survival group than deceased group.

**Table 1:** Basic characteristics at baseline of deceased group and survival group.

	Deceased, N = 16	Survival, N = 13	*P*
Age, year	65.81 ± 7.66	66.62 ± 13.13	.263
Sex, male:female	9:7	8:5	.388
Underlying disease, n(%)	3(18.75)	3(23.08)	.487
Lymphocyte, ×10^9^/L	0.45 ± 0.162	0.60 ± 0.324	.051
Albumin, g/L	29.19 ± 4.15	30.00 ± 5.23	.350
Hemoglobin, g/L	78.44 ± 6.09	77.07 ± 4.70	.514
TEN, n (%)	3(18.75)	10(76.92)	**<.001** *

TEN = transpyloric enteral nutrition.

* *P* < .001, comparison between deceased group and survival group.

### 3.2. Analysis of the clinic outcomes in TEN group and gastrogavage group

As shown in Table 2, there was no difference in age and sex in TEN group and gastrogavage group. The serum level of albumin at 5th day after the admission in TEN group was significantly higher than gastrogavage group, no difference was found in serum level of albumin at 3rd day after the admission between two groups. In addition, APACHE II score and frequency of feeding suspension in TEN group was lower than gastrogavage group. We also observed that TEN group had lower incidence of abdominal distension and gastric retention than gastrogavage group (Table 3). All these findings indicated that COVID-19 patients in ICU on TEN had better clinic outcomes than those on gastrogavage.

**Table 2:** Comparison of clinic characteristics and outcomes between TEN group and gastrogavage group.

	TEN, N = 13	Gastrogavage, N = 16	*P*
Age, year	66.23 ± 12.62	66.13 ± 8.33	.979
Sex, male: female	9 (69.23)	10 (62.50)	.323
3rd day after admission			
Lymphocyte, ×10^9^/L	0.48 ± 0.237	0.45 ± 0.252	.739
Albumin, g/L	35.64 ± 4.65	31.94 ± 5.42	.077
Hemoglobin, g/L	7.55 ± 0.46	7.57 ± 0.68	.947
APACHE II	20.00 ± 2.23	21.25 ± 2.84	.207
5th day after admission			
Lymphocyte, ×10^9^/L	0.32 ± 0.12	0.41 ± 0.28	.339
Albumin, g/L	38.84 ± 6.12	33.41 ± 4.60	**.029** *
Hemoglobin, g/L	7.41 ± 0.51	7.53 ± 0.59	.557
APACHE II	7.46 ± 1.61	10.63 ± 3.40	**.003** **
Frequency of feeding suspension	2.46 ± 0.88	3.82 ± 1.61	**.011** *
Death, n (%)	5 (38.46)	11 (68.75)	**<.001** ***

APACHE II = Acute Physiology and Chronic Health Evaluation II, TEN = transpyloric enteral nutrition.

* *P* < .05,

** *P* < .01,

*** *P* < .001, comparison between TEN group and gastrogavage group.

**Table 3:** Comparison of the incidence of nutritional intolerance between TEN group and gastrogavage group.

	Abdominal distension, n (%)	Nausea/vomiting, n (%)	Gastric retention, n (%)	Diarrhoea n (%)	Gastrointestinal bleeding, n (%)
TEN, N = 13	1 (7.70)	1 (7.70)	2 (15.38)	2 (15.38)	1 (7.70)
Gastrogavage N = 16	4 (25.00)	2 (12.50)	5 (31.25)	3 (18.75)	1 (6.25)
*P*	**.001** *	.259	**.007** **	.451	.579

TEN = transpyloric enteral nutrition.

* *P* < .05,

** *P* < .01, comparison between TEN group and gastrogavage group.

### 3.3. Dynamic changes of inflammatory cytokines in patients with COVID-19 in ICU

**Figure 1.**
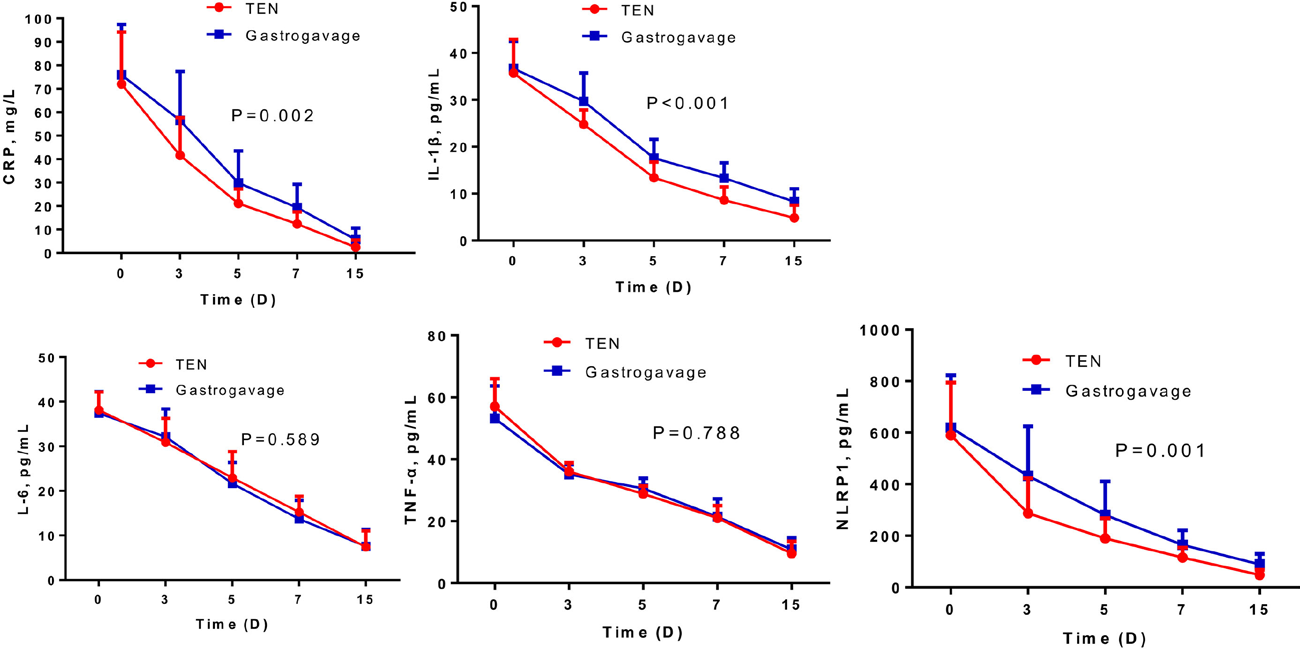
Dynamic changes of inflammatory cytokines in COVID-19 patients. COVID-19 = Corona Virus Disease-19.

We further detected the changes of inflammatory cytokines in all patients. The results showed that serum levels of NLRP1, CRP, IL-1β, IL-6 and TNF-α decreased in a time-dependent manner (Fig. 1). Besides, serum levels of NLRP1, CRP and IL-1β in TEN group was obviously lower than those in gastrogavage group, but no difference was observed in IL-6 and TNF-α between the two groups. A positive correlation was found among serum NLRP1 and inflammatory mediators (IL-6, IL-1β and TNF-α) on 3rd day, and inflammatory mediators (CRP, IL-1β and TNF-α) on 7rd day after the admission (Tables 4 and 5). These results suggested that TEN could notably attenuate inflammatory response than gastrogavage for COVID-19 patients in ICU.

**Table 4:** Pearson correlation analysis for NLRP1 and other inflammatory mediators on 3rd day after admission.

		IL-6	CRP	IL-1β	TNF-α
NLRP1	Pearson correlation	0.430	0.204	0.422	0.512
	*P*	.020	.289	.022	.005

IL-6 = interleukin-6, CRP = C-reactive protein, IL-1β = interleukin-1β, NLRP1 = NLR family pyrin domain containing 1, TNF-α = tumor necrosis factor-α.

**Table 5:** Pearson correlation analysis for NLRP1 and other inflammatory mediators on 7th day after admission.

		IL-6	CRP	IL-1β	TNF-α
NLRP1	Pearson correlation	0.251	0.780	0.535	0.538
	*P*	.190	<.001	.003	.003

IL-6 = interleukin-6, CRP = C-reactive protein, IL-1β = interleukin-1β, NLRP1 = NLR family pyrin domain containing 1, TNF-α = tumor necrosis factor-α.

### 3.4. Diagnostic value of serum NLRP1 for the death of patients with COVID-19

**Figure 2.**
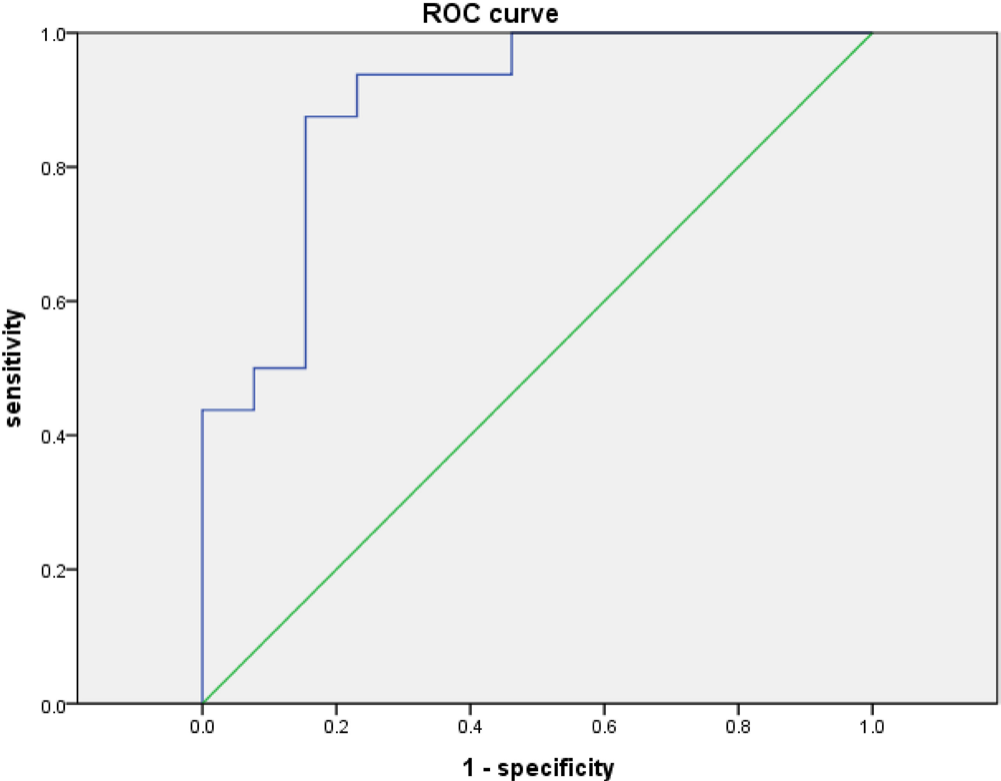
ROC curve for the diagnostic value of NLRP1 for the death of COVID-19 patients. COVID-19 = Corona Virus Disease-19, NLRP1 = NLR family pyrin domain containing 1, ROC = receiver operating characteristic.

The diagnostic value of serum NLRP1 for the death of patients with COVID-19 was analyzed. The data suggested the cutoff value of NLRP1 was 544.50 pg/mL, with AUC of 0.894, sensitivity of 87.5% of and specificity of 84.6%. Receiver operating characteristic curve indicated that NLRP1 might be a potential diagnostic biomarker for the death of COVID-19 patients (Fig. 2).

### 3.5. Comparison of 3-month survival time between patients with low NLRP1 and high NLRP1

**Figure 3.**
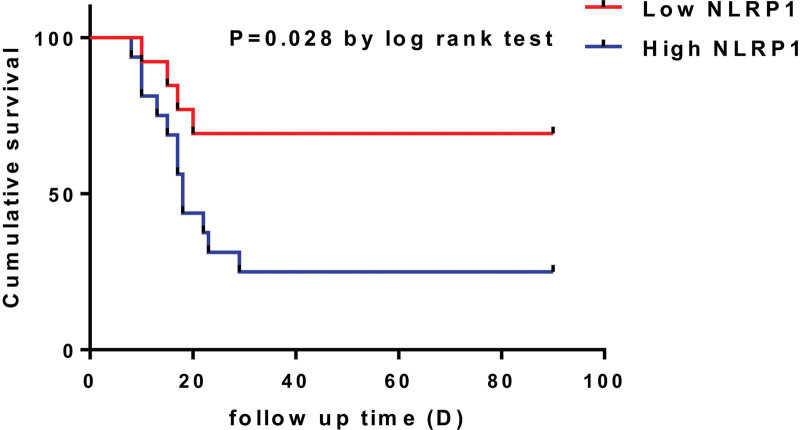
K-M curve analysis for cumulative survival time for COVID-19 patients in high/low NLRP1 group. COVID-19 = Corona Virus Disease-19, K-M curve = Kaplan-Meier curve, NLRP1 = NLR family pyrin domain containing 1.

All the COVID-19 patients were followed up for three months. The mortality rate of low NLRP1 group was 30.8% (4/13) and high NLRP1 group was 75.0 % (12/16). K‐M curve suggested that patients with low NLRP1 expression had longer survival time during the follow-up (*P* = .028, Fig. 3).

## 4. Discussion

Up to 80% of the COVID-19 patients show mild to moderate severity of pulmonary inflammation while the rest might develop into severe pneumonia with poor prognosis.^[15]^ Severe patients consume a lot of energy and need more protein, if the nutrition supplement is not timely and inadequate, it will affect the immune function and increase the incidence of complications, resulting in reduced efficacy of treatment and even poor prognosis.^[16]^ Immune system is interacted with nutrients across the life course.^[17]^ Athletes with a well-balanced diet is sufficient to maintain immune function, meanwhile, inadequate or inappropriate nutrition will induce negative influence on immunocompetence.^[18]^ As we know, adequate substrates of energy, protein, carbohydrates, lipids, vitamins and minerals are of great significance to maintain health.^[19]^ Malnutrition is a common feature for most severe patients. As reported, malnutrition is positively correlated with the length of stay and a greater risk of complications in surgical patients with gastrointestinal cancer.^[20]^ A previous study also shows that nutrition risk score is identified as a prognostic factor for the development of complications with odds ratios of 4.2, besides; nutrition risk score, nutrition risk index, and bioimpedance analysis were associated with the incidence and severity of perioperative complications in gastrointestinal surgery.^[21]^ However, up to now, no researches focused on clinic effect of TEN on inflammatory response and prognosis of COVID-19 patients in ICU. In the present study, we demonstrated that TEN could significantly decrease the serum level of CRP and IL-1β. Critical ICU patients who received TEN treatments had higher level of albumin and lower APACHE II score, frequency of feeding suspension, mortality and incidence of nutritional intolerance than gastrogavage. However, there was no difference for 3-month survival time between patients on TEN and patients on gastrogavage.

TEN has been identified as a safe and effective way of enteral nutrition that prevents from gastric retention and reduces the incidence of gastrointestinal complications and aspiration pneumonia.^[22]^ As reported, TEN was a useful and simple feeding method that enables a high calorie delivery to be provided with few complications in the postoperative period of cardiac surgery in children. Transpyloric feeding had good tolerance to nutrition and was not affected by the infusion of vasoactive drugs, sedatives, or muscle relaxants.^[23]^ A review also revealed that postpyloric enteral nutrition was suitable children with a high risk of aspiration or when gastric feeding either was contraindicated or had failed.^[24]^ A study on tolerance to enteral nutrition in the critically ill child with shock also illustrated that most critically ill children with shock can tolerate postpyloric enteral nutrition, but the incidence of gastrointestinal complications was higher.^[25]^ However, no researches demonstrated the clinic outcomes of TEN on ICU patients with COVID-19. This study found that TEN could decrease rate of mortality, disease severity and increase serum albumin level. What’s more, there was no difference for 3-month survival time between ICU patients with COVID-19 on TEN and gastrogavage.

Emerging study showed that COVID-19 infection is accompanied by an aggressive inflammatory response with the release of a large amount of pro-inflammatory cytokines, followed by lung injury, multiorgan failure, and unfavorable prognosis of severe COVID-19.^[26–28]^ Predictive biomarkers of pathogenic inflammation could evaluate the disease severity of patients with COVID-19. CRP levels were reported to be positively correlated with the diameter of lung lesion and severe presentation, and CRP level reflected disease severity in the early stage of COVID-19.^[29]^ A meta-analysis also found that in nonsevere COVID-19 patients had lower levels for CRP, PCT and IL-6, in addition, survivors had a lower level of IL-6 than the deceased.^[30]^ The anti-inflammatory effects of enteral nutrition (EN) was reported in the study of Bannerjee et al, for patients on EN, numerous inflammatory markers including sedimentation rate, CRP and IL-6 was quickly decreased to be normal level before any detectable improvement in nutrition status.^[31]^ In this study, we also notice that TEN therapy significantly decreased serum levels of NLRP1, CRP and IL-1β.

NLRP1 was confirmed as a proinflammatory protein in various diseases. As a kind of inflammasome sensors, NLRP1 mutations contributed to the activation of inflammasome in human skin.^[32]^ Upregulated NLRP1 was associated with the increase of IFN-γ in ulcerative colitis and accelerated the progression of inflammatory bowel disease.^[33]^ NLRP1 was also involved in the development of lung diseases. A recent study revealed NLRP1 was a key sensor of SARS-CoV-2 infection in lung epithelia.^[34]^ NLRP1 assembly enhanced release of caspase-1 and IL-1β and the activation of NLRP1 inflammasome promoted cell death and induced acute lung injury in mice.^[35]^ Our study showed that serum NLRP1 was positively correlated with CRP, IL-6, IL-1β and TNF-α in COVID-19 patients. Besides, we for the first-time revealed serum NLRP1 might serve as a promising diagnostic biomarker for the death of COVID-19 patients, and those with lower NLRP1 had longer survival time.

This study also has some limitations. The sample size is small. Besides, this is a prospective study, and it is urgent to further confirm the conclusion according to the principle of randomized double-blind control. Finally, the deep investigation is needed for the molecular mechanism of NLRP1 in COVID-19 patients.

## 5. Conclusion

In summary, this observational study found that TEN significantly improved prognosis and attenuates inflammatory response than gastrogavage for COVID-19 patients in ICU. Besides, NLRP1 exhibited valuable potential for the diagnosis of the death of COVID-19 patients, and those with higher NLRP1 had shorter survival time. This study might provide clinical evidence for the effect of TEN on NLRP1, inflammation and prognosis for COVID-19 patients in ICU.

## Author contributions

**Writing – original draft:** Ke Wang, Ping Chen, Wei Zhu, Xiaolei Xing.
